# Developmental Characteristics and Auxin Response of Epiphytic Root in *Dendrobium catenatum*

**DOI:** 10.3389/fpls.2022.935540

**Published:** 2022-06-23

**Authors:** Jili Tian, Weiwei Jiang, Jinping Si, Zhigang Han, Cong Li, Donghong Chen

**Affiliations:** State Key Laboratory of Subtropical Silviculture, Zhejiang A&F University, Hangzhou, China

**Keywords:** *Dendrobium catenatum*, epiphytic life, aerial root, auxin response, DcWOX12

## Abstract

Dendrobium catenatum, a traditional precious Chinese herbal medicine, belongs to epiphytic orchids. Its special life mode leads to the specialization of roots, but there is a lack of systematic research. The aerial root in *D. catenatum* displays diverse unique biological characteristics, and it initially originates from the opposite pole of the shoot meristem within the protocorm. The root development of *D. catenatum* is not only regulated by internal cues but also adjusts accordingly with the change in growth environments. *D. catenatum* root is highly tolerant to auxin, which may be closely related to its epiphytic life. Exogenous auxin treatment has dual effects on *D. catenatum* roots: relatively low concentration promotes root elongation, which is related to the induced expression of cell wall synthesis genes; excessive concentration inhibits the differentiation of velamen and exodermis and promotes the overproliferation of cortical cells, which is related to the significant upregulation of WOX11-WOX5 regeneration pathway genes and cell division regulatory genes. Overexpression of *D. catenatum* WOX12 (DcWOX12) in Arabidopsis inhibits cell and organ differentiation, but induces cell dedifferentiation and callus production. Therefore, DcWOX12 not only retains the characteristics of ancestors as stem cell regulators, but also obtains stronger cell fate transformation ability than homologous genes of other species. These findings suggest that the aerial root of *D. catenatum* evolves special structure and developmental characteristics to adapt to epiphytic life, providing insight into ideal root structure breeding of simulated natural cultivation in *D. catenatum* and a novel target gene for improving the efficiency of monocot plant transformation.

## Introduction

**FIGURE 1:**
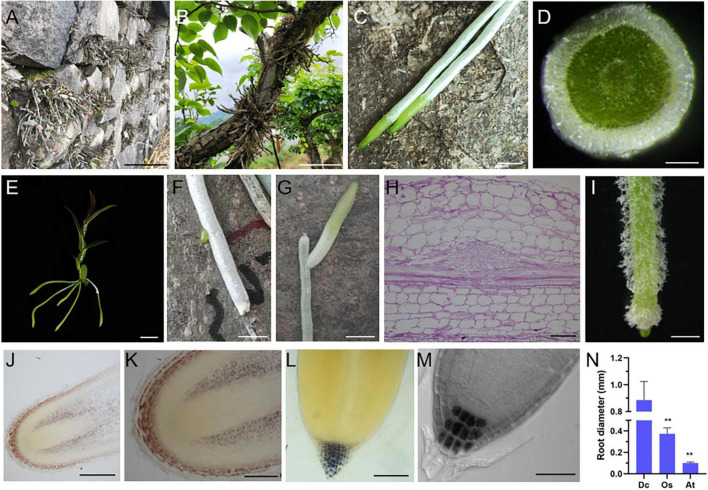
Unique biological characteristics of *D. catenatum* root. **(A)** Rock epiphytic mode, **(B,C)** trunk epiphytic mode showing the aerial roots attached to a living tree **(C)**; **(D)** manual cross-section of an aerial root; **(E)** a plantlet of *D. catenatum* is absent of lateral roots. **(F,G)** lateral root production near the injury site; **(H)** paraffin section of lateral root primordia displayed by PAS staining; **(I)** root hairs from a tissue culture *D. catenatum* plantlet. **(J–L)** Iodine staining of roots in seedlings of *D. catenatum* **(J,K)**, rice **(L)**, and Arabidopsis **(M)**; **(N)** comparison of root diameters among *D. catenatum*, rice, and Arabidopsis. Dc, *D. catenatum*; Os, *Oryza sativa*; At, *Arabidopsis thaliana*. **p < 0.01 (two-sample *t-*test, compared with Dc). Bars = 5 cm in **(A,B)**, 0.25 cm in **(C**,**F,G)**, 250 μm in **(D,J)**, 1 cm in **(E)**, 50 μm in **(H)**, 1 mm in **(I)**,100 μm in **(K,L)**, 25 μm in **(M)**.

*Dendrobium catenatum* is a traditional rare and precious Chinese medicinal material, which was first described in Shennong’s Classic of Materia Medica (Si et al., 2017a,b). Modern pharmacology has confirmed that *D. catenatum* has a wide range of effects, such as enhancing immunity, anti-cancer, protecting liver and stomach, lowering blood sugar, lipid, and blood pressure, anti-fatigue, and anti-oxidation (Si et al., 2017a). Since the 1990s, with the breakthrough of key industrial technologies such as artificial pollination, tissue culture rapid propagation, and cultivation substrate selection, *D. catenatum* resources have changed from wild sporadic mining to large-scale facility cultivation, resulting in the transformation of *D. catenatum* from rare and endangered plants into bulk medicinal materials and formation of 10 billion level of industry (Si et al., 2017a). The yield of *D. catenatum* under facility cultivation is high, but the content of functional components is lower than that in the wild. Therefore, the simulated natural cultivation modes of *D. catenatum*, such as “living tree epiphytic mode” and “lithophytic cultivation,” which take both yield and quality into consideration, came into being (Figure 1). Root is closely associated with the epiphytic life of *D. catenatum* in simulated natural cultivation, and investigation on the development and regulation of root is helpful to the breeding of ideal root structure and the guidance of the production practice.

The roots of epiphytic orchids represented by *D. catenatum* are directly exposed to the air, the acquisition of water and nutrition is irregular, and they often face environmental stresses such as drought and heat. The unique lifestyle of epiphytic orchids led to modified aerial root, which is different from the common root characteristics. Epiphytic orchid roots mostly contain chlorophyll, and they are usually in a photosynthetic dormant state, but rapidly activated when wet and go dormant again when dry (Zotz and Winkler, 2013; Sma-Air and Ritchie, 2020). Though epiphytic orchid root still has the similar tissue pattern as the common root anatomically, the outermost tissue is not a single layer of epidermis, but a spongy multilayered velamen radicum, which is composed of dead epidermal cells at maturity, and is capable of rapidly absorbing water and binding cations with very slow water loss rate (Zotz and Winkler, 2013; Hauber et al., 2020). The outstanding ability of roots to absorb and store water is an important feature of epiphytic orchids to survive in water-limited environments. Moreover, the velamen radicum is also considered to be the adaptive characteristics with multiple roles, such as protection against UV-B radiation (Chomicki et al., 2015), and providing a habitat of microbial symbionts associated with nitrogen fixation and the synthesis of hormones and metabolites related to stress tolerance and growth promotion (Deepthi and Ray, 2018, 2020). In general, there is a lack of systematic understanding of the development and regulation mechanism of epiphytic orchid roots. The unique epiphytic characteristics and public reference genome resources of *D. catenatum* make it very suitable as a model system for the study on epiphytic aerial roots.

Plant root systems include embryonic roots, primary roots, lateral roots (LRs), and adventitious roots (ARs), playing critical functions in fixing, absorption, and storage. In model plant Arabidopsis, embryo roots originate from hypophysis as the uppermost cell of the suspensor at the globular stage during embryogenesis (Chen et al., 2019). During post-embryonic stage, primary roots derive from the continuous growth of embryonic roots and breakthrough of testis, and LRs are developed from LR primordia that are usually endogenously initiated from xylem pole pericycle cell in a developing root. ARs are regenerated from non-root organs (e.g., hypocotyl, stem, or leaf) or very old roots (Ge et al., 2019), which are typically induced and activated by diverse stresses and developmental cues, provide the adaption plasticity to ever-changed environments during the evolution of land plants, and are considered to be a strong natural selective trait (Lardon and Geelen, 2020). ARs mainly arise from pericycle-like cells neighboring vascular tissues and alternatively from phloem or xylem parenchyma cells, young secondary phloem cells, and procambium/cambium cells within vascular tissues (Lakehal and Bellini, 2019). Epiphytic orchids are adventitious root-based and do not make later roots (Shekhar et al., 2019).

Adventitious root is produced from non-root organs upon exogenous and endogenous cues and is a process of *de novo* root regeneration (DNRR) (Xu, 2018). Auxin is the major growth-promoting hormone for AR initiation. Auxin application by suitable concentration is widely used in asexual reproduction (e.g., cutting, air layering, and tissue culture) for DNRR in horticulture and forestry. The regulatory mechanisms of auxin are primarily orchestrated through biosynthesis, conjugation, polar transport, and signal transduction (Xie et al., 2021). For instance, overexpression of auxin receptor TIR1 and transcription factor AUX1 promotes AR development (Street et al., 2016). During DNRR, the regeneration-competent cells (e.g., pericycle-like) in explant experience fate transition guided by auxin (Yu et al., 2017; Xu, 2018). Cell fate transition from regeneration-competent cells to AR formation is an orderly and organized process, further divided into four steps: priming, initiation, patterning, and emergence (Xu, 2018). In addition, a high level of exogenous auxin can result in callus induction, which occurs from pericycle-like cells *via* a root developmental program (Sugimoto et al., 2010), similar to the DNRR (Liu et al., 2014). Both AR and callus regeneration events involve two cell fate transition steps: priming step from regeneration-competent cells to root founder cells (marked by WOX11/12), and initiation step from founder cells to root primordium or callus with remarkable root primordium-like feature (marked by WOX5), which is maintained by the constantly high endogenous auxin level (Hu and Xu, 2016; Sheng et al., 2017). Auxin distribution in callus mass is uneven, resulting in the entry of some cells with low auxin levels into patterning step in advance and the expression of RAM identity genes. Therefore, callus consists of a group of heterogeneous cells with root primordium features and partially differentiated RAM traits.

Here, we systematically revealed the developmental characteristics, origin, adaptive changes to the environment, and high tolerance and response to auxin of *D. catenatum* roots, providing guidance for production practice, and identified *D. catenatum* WOX12 high-efficient dedifferentiation-inducible property, providing novel candidate gene resource for regeneration and plant transformation.

## Materials and Methods

### Plant Materials and Growth Conditions

The *D. catenatum* cultivars (JP966, JP7869, and D6) and *D. nobile* from Zhejiang A&F University and wildtype Arabidopsis Col-0 were used in this study. For Arabidopsis growth, seeds were surface sterilized (70 and 95% ethanol each for 10 min) and plated on 1/2 MS medium. After stratification at 4°C for 2 days in the dark, plates were incubated in a growth chamber at 22°C under a 16-h light/8-h dark photoperiod.

For *D. catenatum* tissue culture, seedlings were grown in greenhouse at a temperature of 25°C and 16-h light/8-h dark photoperiod. For pine bark pot cultivation and rock/trunk epiphytic cultivation of *D. catenatum*, all samples were grown in Hengtang Medicine-Expo Garden, Tianmu Mountain Eco-Agriculture Demonstration Zones, Hangzhou, Zhejiang Province, China (119°26′11″E, 30°20′30″N, 280 m above sea level).

For *D. catenatum* seed germination, a mature capsule from JP966 cultivar was subjected to surface sterilization including 75% ethanol for 1 min and 2% HgCl_2_ for 10 min and washed by sterilized water for at least 5 times. Subsequently, the seeds from sterilized capsule were sowed on the germination medium (1/2 MS + 0.1 mg/L NAA + 0.5% active carbon + 3% sucrose + 0.8% agar, pH5.8).

For auxin treatment, 6-month-old *D. catenatum* JP966 tissue culture seedlings with the removal of the roots were inoculated to auxin-free banana medium (MS + 10% banana pulp + 3% sucrose + 0.8% agar, pH5.8) containing different concentrations of NAA (0, 2, 5, 10, and 20 mg⋅L^–1^).

For overexpressing *DcWOX12-GFP* in transgenic Arabidopsis, the full-length ORF of *DcWOX12* (LOC110108675) without stop codon was amplified from a cDNA pool of the *D. catenatum* seedlings and inserted into the modified pCAM1300-GFP vector, using the following primers: 5′-ggatccATGGAAGAGGAGAAGCCCCC-3′ and 5′-gtcgacGCTC CGGTTATCACCAAAGAAGT-3′. The construct was then introduced into Col-0 wildtype Arabidopsis plants *via Agrobacterium tumefaciens*-mediated transformation.

### Histology and Microscopy

Vibratome sectioning was performed as described (Henry et al., 2015). Briefly, fresh root samples were directly embedded in 3% agarose blocks and then were glued on a vibratome plate to be sliced. Transverse sections were obtained using a Leica VT1200/VT1200S vibration microtome (Leica, Germany) with speed 0.01–1.5 mm/s, amplitude 0.8 mm, and thickness 50 μm. On the one hand, the obtained slices were used for direct observation or stained with 0.1% aniline blue solution. Lignin staining with phloroglucinol-HCl reagent was performed as previously described (Joca et al., 2020). Starch staining with Lugol’s solution and fat red staining with Fat Red 7B were performed as previously described (Chen et al., 2010). On the other hand, the slices were transferred either onto chamber slides (Lab-teak 177402) for immunolocalization.

Immunolocalization assay was performed as previously described (Henry et al., 2015). Briefly, the sections in the chamber slides experienced the rinse with 0.1 M glycine and phosphate-buffered saline (PBS) solution, blocking with 5% bovine fetal serum, incubation with corresponding the primary and secondary antibodies. Several specific cell wall-directed probes available from PlantProbes^1^ were used as primary antibodies to evaluate the presence of major cell wall polymers including (1→4)-β-galactan (LM5) (Andersen et al., 2016), heteromannan (LM21) (Marcus et al., 2010), and pectin (JIM5, JIM7) (Clausen et al., 2003). Secondary antibodies include Alexa 546 anti-rat antibody (Invitrogen A11081) or Alexa 546 anti-mouse antibody (Invitrogen A11060). Confocal observation was performed with a Zeiss LSM 880 confocal microscope (ZEISS, Germany).

Paraffin sectioning was performed as previously described (Chen et al., 2016), Briefly, *D. catenatum* root samples were fixed with FAA solution for at least 24 h, dehydrated in gradient ethanol/tert-Butyl alcohol solutions, and embedded in paraffin. The paraffin tissue blocks were sectioned to 6 μm by a rotary microtome (Leica, Germany). Then, the paraffin sections were stained by safranin O-fast green staining *via* Safranin O-Fast Green stain kit (Solarbio, China) or PAS staining *via* glycogen periodic acid Schiff (PAS/hematoxylin) stain kit (Solarbio, China).

Differential interference contrast (DIC) observation was performed as previously described (Chen et al., 2016). Plastic sectioning and transmission electron microscopy (TEM) observation were performed as previously described (Yu et al., 2009). All conventional light microscopy was performed with Olympus SZX16 or Olympus BX60 microscopes (Olympus, Japan).

### RNA-Seq and Data Analysis

For auxin treatment, the root samples of *D. catenatum* plantlets treated with different NAA concentrations (0, 5, 10, and 20 mg L^–1^) for 3 months were harvested for RNA-seq. For DcWOX12 function analysis, 2-week-old Arabidopsis seedlings of *DcWOX12-GFP* overexpression transgenic line and Col-0 wildtype were collected for RNA-seq. A total of three independent RNA samples (biological replicates) were performed. RNA was extracted using the MiniBEST Plant RNA Extraction Kit (TaKaRa, Japan) according to the manufacturer’s instructions. The paired-end sequencing was performed on an Illumina HiSeq 4000. Reads of above-described samples were aligned to the genomes *D. catenatum* or Arabidopsis using HISAT package (Kim et al., 2015), which initially remove a portion of the reads based on quality information accompanying each read and then maps the clean reads to the reference genome. StringTie was used to assemble the mapped reads of each sample and estimate the expression levels of all genes by calculating FPKM (Pertea et al., 2015). The differentially expressed genes were defined with | log2(fold change)| ≥ 1 and with an adjusted *p*-value (*q*-value) < 0.05 by R package (Anders and Huber, 2010). Heatmap was generated using TBtools software (Chen et al., 2020). Differentially expressed genes (DEGs) by auxin treatments were clustered with STEM (Ernst and Bar-Joseph, 2006) based on OmicShare platform for data analysis^2^. Gene Ontology (GO) enrichment in DEGs was identified compared to the reference transcriptome background using OmicShare tools.

### Statistical Analysis

All data were statistically analyzed using IBM SPSS Statistics 26 software and presented as the mean ± SE. Independent-samples *t*-test was used to calculate significant differences between two groups of data, which are denoted by asterisks (*p < 0.05; ^**^p < 0.01).

## Results

### Unique Biological Characteristics of *D. catenatum* Root

*Dendrobium catenatum* is a typical epiphytic orchid, which usually grows on the surface of forest trunk or cliff in natural environment (Figures 1A,B). Due to its unique lifestyle, the root of *D. catenatum* is highly specialized. When exposed to the air, the root tip (representing meristem zone and transition zone) keeps green, and the rest of root (representing mature zone) looks white (Figure 1C), which is unique to epiphytic orchids. It is caused by the spongy velamen radicum that consists of multilayered epidermal cells, becomes dead at maturity (Figure 1D), and has protection and water storage function. However, the internal tissues are still green (Figure 1D), indicating that the root of *D. catenatum* has nutritional function and can synthesize organic compounds. *D. catenatum* usually grow without lateral roots and root hairs (Figure 1E), which may be due to direct exposure to the air and does not need to increase the contact area with the soil. However, when the root was injured, lateral root primordia would be induced near the wound, and lateral roots would grow to maintain the function of the mother root (Figures 1F–H). When sterilely growing in culture bottle, root hairs can be induced by high humidity to enhance the absorption function of roots (Figure 1I). In the epiphytic mode of *D. catenatum*, the gravitropism of roots is not obvious. It is speculated that the roots in this case adhere to the surface of trunk or rock by secreting special polysaccharides, such as glucomannan. Iodine staining also showed that the entire root cap region of *D. catenatum* contained starch granules, evidently different from the common terrestrial plants including Arabidopsis and rice, in which starch granules were only confined to columella cells in the root cap (Figures 1J–M). In addition, the root of *D. catenatum* was thick and sturdy (Figure 1N), with an average diameter of around 1.2 mm, much thicker than that of rice (∼0.3 mm) or Arabidopsis (∼0.1 mm), and even had starch deposition in cortex parenchyma cells (Figures 1J,K), and these properties lead to structural rigidity and insensitivity to gravity. So, the specialization of morphological characteristics in *D. catenatum* is closely related to its epiphytic life mode.

### Developmental Origin of *D. catenatum* Root

**FIGURE 2:**
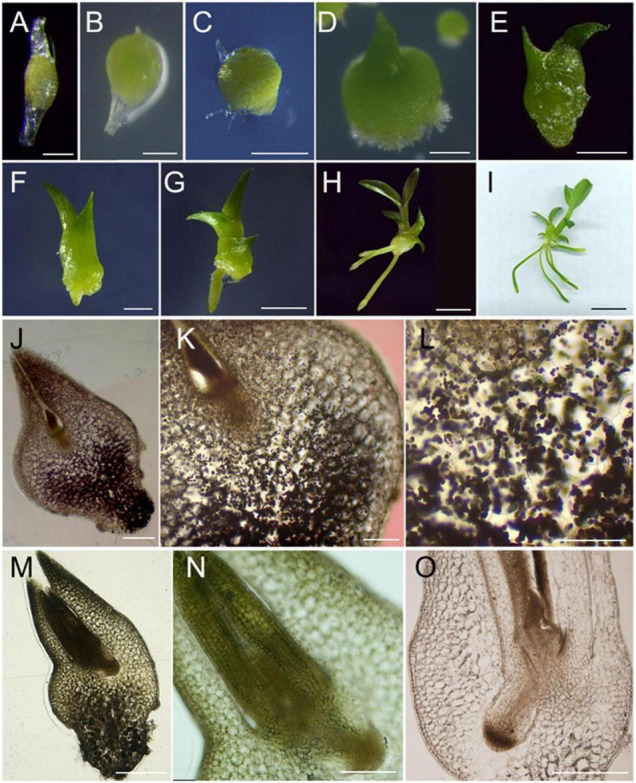
Developmental origin tracing of *D. catenatum* root. **(A–I)** Seed germination and seedling development. **(A)** 0 DAG (day after germination), **(B)** 10 DAG, **(C)** 20 DAG, **(D)** 40 DAG, **(E)** 80 DAG, **(F)** 100 DAG, **(G)** 120 DAG, **(H)** 230 DAG, **(I)** 260 DAG; **(J–O)** Anatomic observation. **(J–L)** 30 DAG, **(M–O)** 40 DAG, **(O)** 80 DAG. Bars = 100 μm in **(A**,**B,K)**, 250 μm in **(C)**, 500 μm in **(D**,**M**,**O)**, 0.1 cm in **(E,F)**, 0.2 cm in **(G)**, 4 cm in **(H)**, 1 cm in **(I)**, 200 μm in **(J,N)**, 50 μm in **(L)**.

Different from the fact that the root cell pattern of the representative model plant Arabidopsis has been completely established during embryogenesis, the embryos in the mature seeds of *D. catenatum* are poorly developed and arrest at a very early stage comparable to the globular stage of Arabidopsis embryogenesis (Fang et al., 2016), resulting in no establishment of polarized embryo axis and no production of radicle. Later on, orchid embryos continue to develop *in vitro* without following traditional embryogenesis procedures, and instead proliferate to form a unique protocorm structure that is sometimes considered to be a continuum of zygotic embryogenesis (Jones and Tisserat, 1990), but is actually distinct from zygotic embryonic tissue at molecular level (Fang et al., 2016). To trace the embryonic origin of *D. catenatum* root, we defined the developmental stages during seed germination based on the landmark developmental events. At first, the embryo in the tiny seed (Figure 2A) experienced morphologically apolar enlargement, resulting in rupture of the testa and protocorm formation (stage I, Figure 2B). Subsequently, appearance of the rhizoid leads to establishment of morphological polarity (stage II, Figure 2C). The apical shoot enclosed by coleoptile was developed (stage III, Figure 2D), and stage IV is the characteristics of the appearance of the first true leaf due to separate from the unfolded coleoptile (Figure 2E). Then, the first adventitious root occurred from the base (stage V, Figure 2F), indicating the developmental phase from protocorm to plantlet. The secondary true leaf appeared, suggesting the stem node formation (stage VI, Figure 2G). As more roots and leaves appear, tillering occurred (stage VII, Figures 2H,I). Further cytological observation (Figures 2J–O) found that during the polarity establishment of the protocorm, shoot meristem surrounding by young leaves (named shoot body) was first generated at the shoot pole, and then, root meristem was generated at the opposite pole, where root protruded besides the base of protocorm. Protocorm seems to be a temporary and compensatory organ that appears when orchid seeds lack endosperm or cotyledon to supply energy for germination. It can not only effectively protect shoot body, but also accumulate starch granules, provide habitat for germination promoting endophytes, and produce rhizoids to absorb certain nutrients from the outside. Therefore, the root of *D. catenatum* originates endogenously from the root meristem opposite to shoot body enclosed by the protocorm.

### Cytological Changes in *D. catenatum* Root Structure During Growth and Development

**FIGURE 3:**
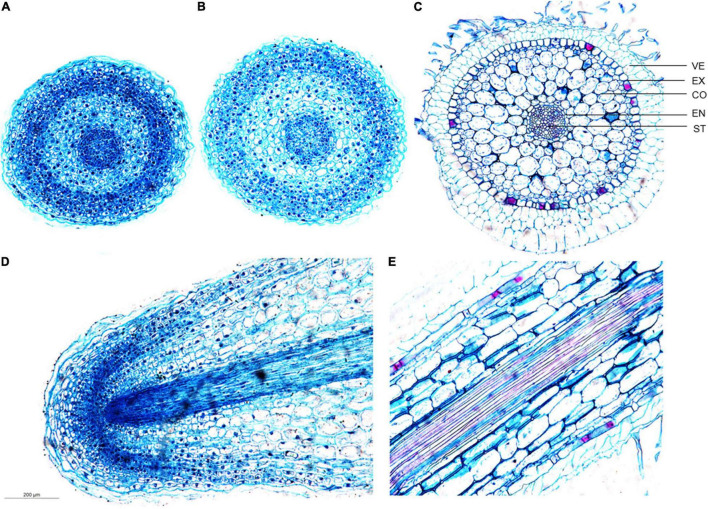
Developmental differences of *D. catenatum* root structure. **(A)** Cross-section of the meristem zone, **(B)** cross-section of the transition zone, **(C)** cross-section of the mature zone, **(D)** longitudinal view of the root apex, **(E)** longitudinal view of the mature zone. VE, velamen; EX, exodermis; EN, endodermis; ST, stele.

To investigate the cytological changes in *D. catenatum* root during development, we performed the paraffin section on the root tips of JP966 tissue culture seedlings (Figure 3). According to the cross-section observation of different regions in root tip, meristematic zone (Figure 3A) and transition zone (Figure 3B) in a low differentiation level roughly included root cap, epidermis, ground tissue, and vascular bundle without distinct boundaries between adjacent cell types. The epidermis cells are still living and exodermis is undistinguishable due to the lack of obvious thickening Casparian strip, and the cells are smaller with compact arrangement and distinct nuclei. The mature zone (Figure 3C) was clearly divided into velamen, exodermis, cortical parenchyma, endodermis, and stele. The velamen is irregular and consists of multiple layers of dead cells. The exodermis is a single layer of lignified sclerenchyma cells (large) with horseshoe-shaped thickening of the cell wall intercalated by passage cells. The cortex is multilayered, where the cells close to both sides are smaller, and those in the middle are larger, oval in cross-section, rectangular in longitudinal section, with gaps among cells, and inconspicuous nuclei. The endodermis is a single layer of sclerenchyma cells (small) and interspersed with passage cells, and much less developed than exodermis. The stele belongs to radical type, and the structure of the pericycle is not obvious. Longitudinal section showed that the root cap surrounding the RAM is underdeveloped (Figure 3D) and well organized cell pattern in mature zone (Figure 3E).

**FIGURE 4:**
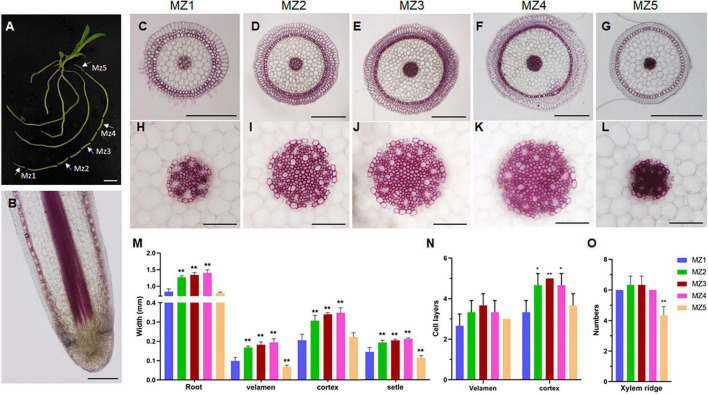
Structural differences of maturation zones during *D. catenatum* root growth. **(A)** Sampling sites of root. White arrows indicated different mature zones (MZ1 to MZ5). **(B)** Longitudinal section of root apex. **(C–L)** Cross-section of mature area of different parts of root. **(H–L)** Steles of different parts of root, Bars = 100 μm. **(M)** The diameter of the root and the width of the velamen, endodermis, and stele. **(N)** The number of cell layers of the velamen and cortex. **(O)** The xylem ridges in different parts. MZ, mature zone. **p < 0.01 (two-sample *t*-test, compared with MZ1). Bars = 1 cm in **(A)**, 25 μm in **(B)**, 500 μm in **(C–G)**, 100 μm in **(H–L)**.

To investigate the change rule of the root structure of *D. catenatum* during growth, we observed different segments of mature zones at the same root of the JP966 tissue culture seedlings by vibratome sectioning and phloroglucinol staining (Figure 4). Phloroglucinol specifically stains lignin, a hallmark component of the secondary cell wall. The lack of staining in the RAM indicates that there is no secondary wall formation at this time (Figure 4B), which is consistent with high cell division activity and low cell differentiation. Overall, the maturation zones at different root segments have similar pattern (Figures 4C–G). However, by comparison of the cross-sections of the representative mature zones (MZs) near the RAM (MZ1), in the middle (MZ2∼4) and near the stem base (MZ5), we found that the diameter of the whole root, velamen, cortex, and stele of different segments of a single root were different (Figures 4C–L). The cross-sectional widths of MZ2/3/4 were significantly larger than those of MZ1 and MZ5 (Figure 4M); correspondingly, the number of cell layers in the velamen and cortex: MZ2/3/4 > MZ1/5 (Figure 4N). The number of xylem ridges was the least in the earliest MZ5 (4), which was the most stained and lignified and then slowly stabilized (7) (Figure 4O). Therefore, the pattern produced by *D. catenatum* root at the beginning of formation (MZ5) is relatively simple and then tends to be stable (MZ3∼5), whereas the newly differentiated mature zone (MZ1) near the root tip is not yet fully differentiated, indicating that structure of the same root varies during growth.

### Immunolocalization of *D. catenatum* Aerial Roots

**FIGURE 5:**
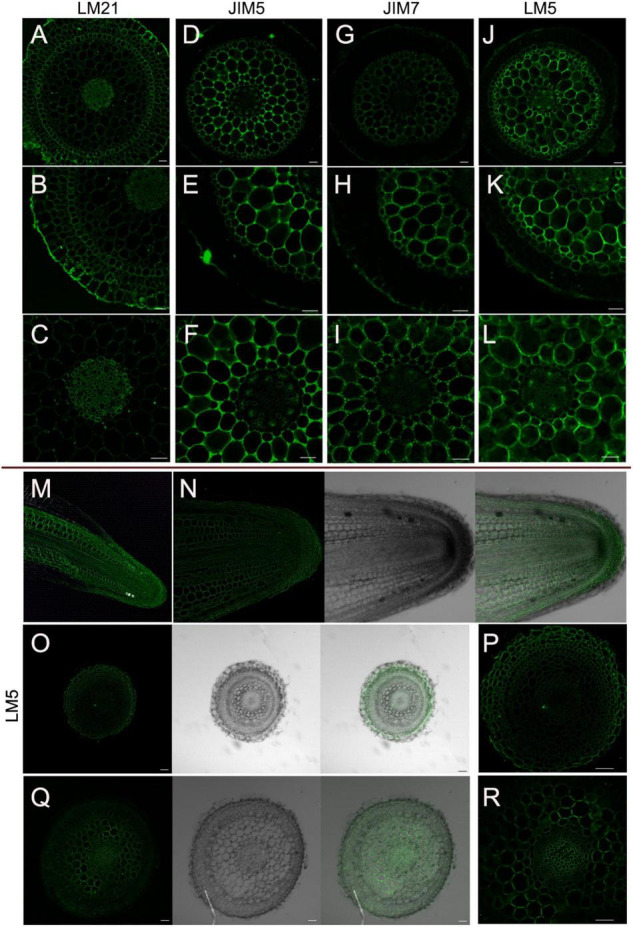
Immunolocalization of *D. catenatum* aerial roots. **(A–L)** Immunolabeling in the mature zone. **(A–C)** LM21 antibody, **(D–F)** LM5 antibody, **(G–I)** JIM5 antibody, **(J–L)** JIM7 antibody. **(M–R)** LM5 epitope in root tip. **(M)** Longitudinal section of root apex, **(N)** longitudinal section of meristem, **(O,P)** transverse section of meristem zone, **(Q,R)** transverse section of transition zone. Bars = 50 μm.

Plant cell wall mainly comprises cellulose, hemicellulose (e.g., xyloglucans, xylans, mixed-linkage glucans, and mannans) and pectin [e.g., homogalacturonan (HG), rhamnogalacturonan-I (RG-I), and RG-II] (Verhertbruggen et al., 2009; Marcus et al., 2010). To identify whether the chemical composition has changed in the specialized *D. catenatum* root or not, specific polysaccharide molecular probes were used for detection (Figure 5). In mature zone, anti-mannan antibody LM21 recognizes the β-linked mannan polysaccharide (Marcus et al., 2010), which was localized on the cell wall of all root cell types of *D. catenatum* (Figures 5A–C). Anti-pectin antibodies JIM5 and JIM7, respectively, recognize low and high levels of methyl-esterified HGs (Willats et al., 2000; Clausen et al., 2003; Henry et al., 2021) and had similar immunolabeling pattern in cortex and stele, and no labeling in velamen, exodermis, and endodermis (Figures 5D–I). The difference was that JIM5 had a strong signal in the cortex tricellular junction delta region (Figure 5F), whereas JIM7 had the strong labeling in the vertices of this delta region (Figure 5I), where high methyl-esterified HGs have a strong ability to form gels in the cell wall matrix, increase cell wall porosity, and might facilitate the transport of solutions among cortical cells (Joca et al., 2020). Anti-pectin probe LM5 recognizes (1-4)-β-D-galactan side chains of RG-I (Andersen et al., 2016) and had strong signal in cortex and phloem, moderate signal in exodermis, endodermis, and stele (excluding phloem), but no signal in velamen (Figures 5J–L). In addition, LM5 epitope had labeling in the undifferentiated cells of meristem zone and transition zone, but no labeling in the root cap in the root tip (Figures 5M–R). Therefore, the content and domain of pectic polysaccharides are closely related to the root tissue types and developmental status.

### Effects of Growth Environment and Germplasm on Root Development

**FIGURE 6:**
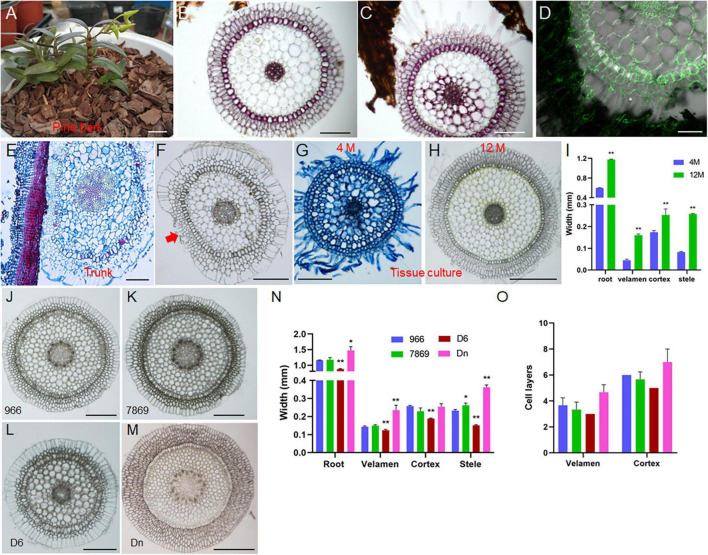
Effects of growth environment and germplasm on root development of *D. catenatum*. **(A–D)** cultivation of *D. catenatum* on bark substrate, **(B)** cross-section of roots away from bark, **(C)** roots close to bark, **(D)** LM5 marker; **(E,F)** cross-cutting of *D. catenatum* root attached to the trunk; red arrow indicated the attachment site. **(G–I)** root cross-cutting of tissue cultured *D. catenatum*, **(G)** 100 D, **(H)** 300 D; **p < 0.01 (two-sample *t*-test, compared with 4M). **(J–O)** Cytological observation of aerial root **(J–M)** and corresponding statistical data **(N,O)** of *D. catenatum* varieties JP966, JP7869, D6, and *D. nobile* (Dn). *P < 0.05 and **p < 0.01 (two-sample *t*-test, compared with JP966). Bars = 1 cm in **(A)**; 200 μm in **(B,C,E–G,J–L)**; 100 μm in **(D)**, 500 μm in **(H,M)**.

To explore the effects of different growth environments on the root development of *D. catenatum*, we compared the root structure grown in distinct conditions (Figure 6). Under pot cultivation mode using pine bark substrate (Figure 6A), root hair was absent on the root surface far away from the substrate (Figure 6B), similar to root status stretching freely in the air; the velamen cells close to the substrate but not in contact may produce root hairs (Figures 6C,D), and the cell wall of velamen and root hair displayed LM5 labeling, indicating that they are living cells; the root closely attached to the pine bark had small and dense cells at the attachment site (Figure 6C), which is similar to the specific velamen cells attached to the truck surface (Figures 6E,F). It is speculated that they are living cells, responsible for plant attachment under tree/rock epiphytic modes. Under the condition of tissue culture (Figures 6F–H), the roots from 4-month-old seedlings displayed many root hairs, but half of the thickness of those from 12-month-old plants. The thickness of each velamen cell layer growing in the substrates (e.g., pine bark and agar medium) is similar (Figures 6B–H), but the outermost cell layer is about 1–2 times wider than the other layers in the velamen of exposed root (Figure 6F), which may be related to its highly efficient water absorption and retention capacity, and provides mechanical protection for the inner living cells. Therefore, the change in growth environment will lead to the corresponding adjustment of velamen structure to better adapt to the diverse environments.

To identify the germplasm on the root development of *D. catenatum*, we compared the root structures exposed to the air in three *D. catenatum* variants (JP966, JP7869, and D6) with *D. nobile* as a reference (Figures 6J–O). The diameter of aerial roots and the width of velamen, cortex, and stele were the largest (∼ 1.5 mm) in *D. nobile*, followed by JP966 and JP7869, and the smallest in D6. These suggested that root cell pattern can serve as an indicator to distinguish different germplasms and species.

### Effects of Auxin on Root Development of *D. catenatum*

**FIGURE 7:**
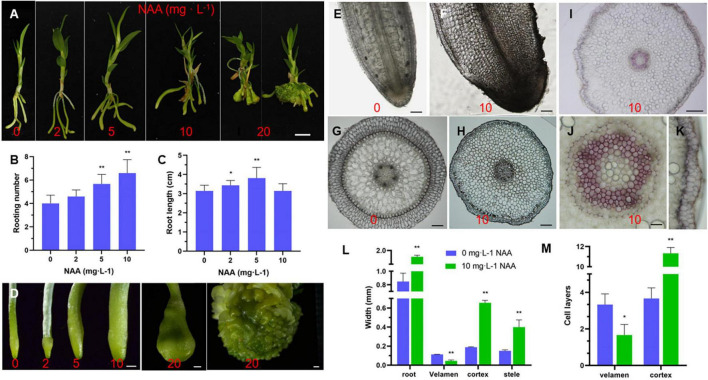
Effect of auxin on root development of *D. catenatum*. **(A–D)** Effect of auxin gradient on phenotype of *D. catenatum*. **(A)** Statistical data of rooting number and root length **(B,C)**, close-up view of roots by gradient auxin treatment **(D)**; **(E–M)** cytological analysis of root thickening caused by high concentration of auxin (10 mg L^– 1^). **(E–H)** Optical observation of vibrating section, **(I–K)** Phloroglucinol staining, **(L,M)** statistical data of thickness and cell layers of different root tissues. *p < 0.05 and **p < 0.01 (two-sample *t*-test, compared with 0 mg L^– 1^). Bars = 1 cm in **(A)**, 1 mm in **(D)**, 200 μm in **(E**,**F**,**H)**,10 μm in **(I)**, 100 μm in **(G)**, 50 μm in **(J,K)**.

During *Dendrobium* seedling growth and propagation, 0.1–2 mg⋅L^–1^ NAA is usually added to the medium during aseptic culture (da Silva et al., 2015). Auxin-producing endophytes in the velamen roots are beneficial for the root colonization of epiphytic orchids (Faria et al., 2013; Pavlova et al., 2017; Shah et al., 2018, 2021, 2022; Deepthi and Ray, 2019; Chand et al., 2020). To explore the effect of auxin on the root development in *D. catenatum*, we transferred JP966 seedlings with the removal of roots to the medium containing different concentrations of NAA (0, 2, 5, 10, and 20 mg L^–1^), the phenotype was observed and recorded after 90 days of cultivation (Figure 7). We found that auxin treatment had two effects: when NAA concentration was lower than 5 mg L^–1^, the plant biomass, root length, and rooting number of *D. catenatum* seedlings were positively correlated with auxin concentration compared with the control (0 mg L^–1^ NAA). Under the condition of high concentrations of NAA (>5 mg L^–1^), with the increase of auxin concentration, the root became shorter, but significantly thicker, and even formed tumor-like structures under the treatment of 20 mg L^–1^ NAA (Figures 7A–D). Cytological analysis further revealed that high concentration of exogenous auxin (10 mg L^–1^ NAA) significantly increased the cortical cell number (∼ 11 layers), compared with ∼ 4 layers in the control, but decreased the width (∼ 0.06 mm) and cell layers (∼2) of velamen, compared with ∼ 0.2 mm width and 3 cell layers in the control, respectively (Figures 7E–M). The exodermis under 10 mg L^–1^ NAA treatment also had smaller cells with significantly decreased lignin staining (Figures 7I–J). Therefore, high concentration of auxin will cause the rapid proliferation of cortical parenchyma cells and inhibit the differentiation of velamen and exodermis.

### Transcriptional Response of *D. catenatum* Root to Auxin Treatment

**FIGURE 8:**
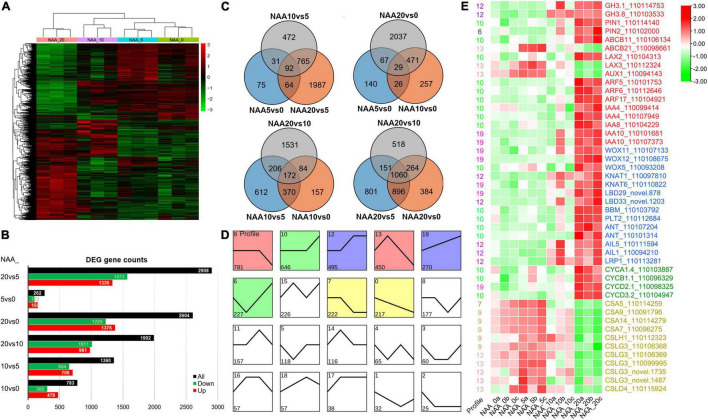
DEGs in different comparisons. **(A)** Cluster of DEGs in different comparisons. Red indicates high expression genes, whereas green indicates low expression genes. **(B)** Statistics of all, downregulated, and upregulated DEGs in different comparisons. **(C)** Venn diagram of DEGs in different comparisons. All DEGs are clustered into three comparison groups represented by three ellipses. The overlapping parts of different ellipses represent the number of DEGs in common from those comparison groups. **(D)** A number of 20 expression profiles of DEGs. **(E)** The heatmap of important DEGs associated with auxin response, stem cell control, cell division, and cell wall synthesis. Red indicates high expression genes, whereas green indicates low expression genes. The FPKM values in each row were processed by *Z*-score normalization.

To analyze the transcriptional response of *D. catenatum* by auxin application, root samples from four NAA treatments (0, 5, 10, and 20 mg L^–1^) were collected for RNA-seq analysis. Gene expression level in each sample was calculated and normalized to FPKM. The differentially expressed genes (DEGs) among these four NAA treatment groups were identified by a false discovery rate (FDR) < 0.05 and a | log2(fold change)| of ≥1. There was a total of 4,448 DEGs among comparison of four treatments (Figures 8A–C).

To further investigate the expression patterns and functions of DEGs among these auxin treatments, the DEGs were clustered by STEM (Ernst and Bar-Joseph, 2006). The total 4,436 DEGs were divided into 20 profiles (Figure 8D). A total of 3,308 (74.57%) of these DEGs were clustered into 8 profiles (*p*-value < 0.05), which included a flat-downward trend for profile 9 (gene number 781), a flat-upward trend (profile 10, 646), a flat-upward-flat trend (profile 12, 495), a flat-downward-flat trend (profile 7, 222), an upward–downward trend (profile 13, 450), a downward–upward trend (profile 6, 227), an upward trend (profile 19, 270), and a downward trend (profile 0, 217).

Since 5 mg L^–1^ NAA significantly promoted root elongation growth, we intersected the differentially upregulated genes (103) in the NAA_5vs0 comparison group with the significantly enriched profiles of trend analysis and found that the genes of interest were concentrated in profiles 9 and 13. Among them, auxin transporters *ABCB21*, *LAX2*, and *LAX3* were significantly upregulated, and cellulose synthase (*CES*) and cellulose synthase-like (*CSL*) genes were significantly enriched (Figure 8E), indicating that relatively low level of auxin promoted root elongation by enhancing the expression of cell wall synthesis related genes. Since high level of auxin (20 mg L^–1^ NAA) significantly promoted root cell proliferation, we intersected the differentially upregulated genes (1,573) in the NAA_20vs5 comparison group with the significantly enriched profiles of trend analysis and found that the genes of interest were concentrated in profiles 10, 12, 19, and 6. Among them, auxin-related genes including conjugating (*GH3.1*, *GH3.8*), transportation (*PIN1*/*2*, *LAX2*), and signal transduction pathway (*ARF5*/*6*/*17*, *IAA4*/*8*/*10*) were significantly upregulated, and stem cell control and regeneration pathway-related genes (e.g., *WOX11*/*12*/*5*) and cell division regulatory genes (*CYC A1;4*/*B1;1*/*D2;1*/*D3;2*) were significantly upregulated (Figure 8E), indicating that high level of auxin promotes cell division and excessive proliferation by inducing regeneration pathway genes.

### Functional Analysis of DcWOX12 Gene

**FIGURE 9:**
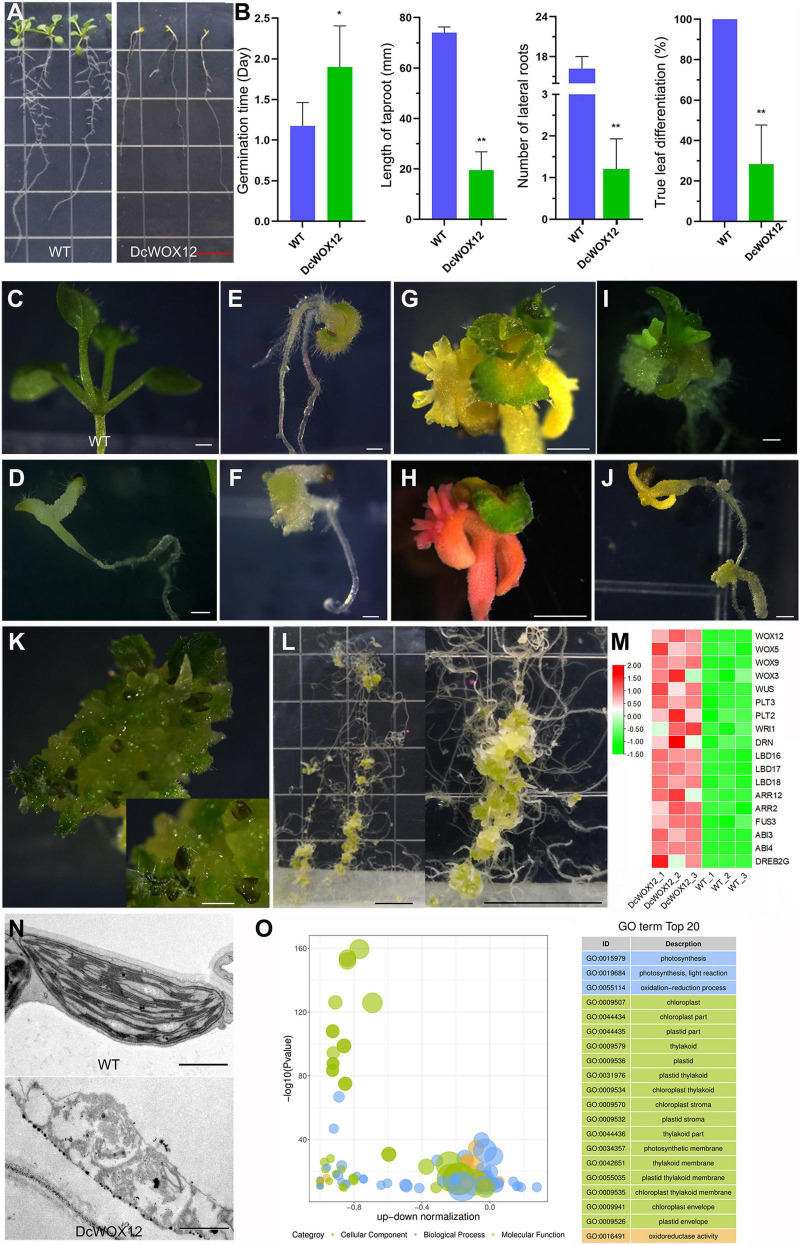
Callus induction by overexpression of *DcWOX12* in Arabidopsis. **(A–L)** The representative phenotype in *DcWOX12* overexpression Arabidopsis; **(A–F)** 2-week-old seedling phenotype, **(B)** showing statistical data of germination time, root length, lateral root number, and true leaf differentiation rate. *p < 0.05 and **p < 0.01 (two-sample *t*-test, compared with 0 mg⋅L^– 1^). **(G–J)** 4W, **(K)** 26W, **(L)** 16W. **(M)** The heatmap of important DEGs associated with callus formation and embryo-like structure. Red indicates high expression genes, whereas green indicates low expression genes. **(N)** Electron micrographs of leaf mesophyll cell. **(O)** Bubble plot of top 20 of GO enrichment of DEGs. The ordinate is –log10 (*p*-value), and the abscissa is the up-down normalization value (the ratio of the difference between the number of upregulated genes and the number of downregulated genes in the total differential genes); the yellow line represents the threshold of *p*-value = 0.05; on the right is the term list with the top 20 *p*-values. Different colors represent different ontology classes. Bars = 1 cm in **(A,L)**; 500 μm in **(C–F)** and **(I–K)**, 1 mm in **(G,H)**, and 1 μm in **(N)**.

Since WOX members are highly induced by high level of auxin (Figure 8E) and WOX11/12 play an important role in root regeneration (Hu and Xu, 2016), we used 35S promoter to drive the expression of a *D. catenatum WOX11/12* homologous gene *DcWOX12* (Supplementary Figure 1) in Arabidopsis for heterologous function analysis (Figure 9A). In *35S*::*DcWOX12* transgenic Arabidopsis, seed germination was delayed 1 day, compared with wild type (Figure 9B). The root growth was also inhibited, and the root length of 2 weeks was ∼ 2 cm, which was much shorter than that of wild type (∼ 7.5 cm); the number of lateral roots in 2 weeks was about 1, which was much lower than that of wild type (∼16) (Figure 9B). More than 70% of the transgenic plants developed slowly, became chlorosis, and failed to form normal true leaves, but there were trichomes on the cotyledons (Figures 9C–F), which reminiscence the characteristics of true leaves. Shoot apexes in some seedlings continued to overproliferate and form a structure-like embryogenic callus, and occasionally differentiated into true leaves (Figures 9G–K); although the cotyledon growth of some seedlings arrested, the roots continued to grow and dedifferentiate to form highly proliferative callus (Figure 9L). Therefore, *DcWOX12* overexpression promotes cell dedifferentiation and cell proliferation and inhibits cell and organ differentiation. Transcriptome sequencing showed that stem cell control, regeneration pathway, and embryo-specific gene expression were significantly increased (Figure 9M). Transmission electron microscopy showed that the chlorosis tissues of *DcWOX12* overexpressing plants failed to develop the typical thylakoid lamella in the chloroplast (Figure 9N). Correspondingly, GO enrichment found that the DEGs related to chloroplast development and photosynthesis pathway were significantly enriched (Figure 9O), which further indicated that DcWOX12 inhibits organ differentiation.

## Discussion

Rooting structure emergence is a key morphological innovation associated with the plant evolutionary adaption from water to land. The earliest rooting structure is rhizoid, which is either unicellular filament in liverwort and hornwort or multicellular in moss (Tam et al., 2015). Fossil evidence indicates that rhizoids associated with bilaterally symmetric axes still serve as the rooting structures in the common ancestor of vascular plants, which should be initially rootless (Hetherington and Dolan, 2018). Subsequently, during the evolution of vascular plants, true roots with vascular tissues arose to provide mechanical support for macrophyte growth and much better ability for absorption and fixation than shallow growing rhizoids. Roots have similar structure and consist of the epidermis, cortex, vascular cylinder, and root cap in distinct species (Fujinami et al., 2020), but phylogenetic analyses together with Devonian fossil evidence have suggested that roots belong to paraphyletic origin and have evolved independently at least two times, characterized by two distinct root-branching events, including apex dichotomous-branching roots in extant lycophytes and endogenously pericycle/endodermis-derived LRs/ARs in euphyllophytes (ferns and seed plants) (Kenrick and Strullu-Derrien, 2014). The emergence and diversification of LRs/ARs greatly enhanced the environmental adaptability and successful land colonization of plants. *D. catenatum* as a typical epiphytic orchid represents a novel plant lifestyle transformation from soil growth to tree/aerial growth, and its root system is directly exposed to severe environmental fluctuations, resulting in further specialization of root developmental program. The most significant change is the appearance of a spongy multilayered velamen, which has strong water absorption and retention capability with great possibility to protect roots from being dehydrated and dying when exposed to the air. Moreover, velamen root cells of epiphytic orchids may harbor phototrophic algal associates inside, such as the cyanobacterial-symbiosis supplying host plants with fixed nitrogen (Tsavkelova et al., 2003a,b; Deepthi and Ray, 2020). There are chloroplasts in the root interior, which can synthesize organic matter and store starch, and enhance the osmotic regulation ability of roots. Due to no need to contact the soil to expand the absorption area, lateral roots and root hairs are usually absent. The starch grains in the whole root cap are evenly distributed, and the gravitropism is not obvious, which is consistent with the epiphytic life of aerial roots. Orchid seeds lacking endosperm are very tiny and easy to spread to the trunk or cliff with the wind; they contain underdeveloped embryo arrested at the globular stage without radicle differentiation (Fang et al., 2016). Germination of orchid seeds is not a spontaneous process and requires symbiosis with specific fungi in the wild (Teixeira da Silva et al., 2015). Upon germination, they do not continue to complete the embryogenesis, but produce a unique transitional organ – protocorm, the interior of which first differentiates into the “shoot body” with SAM and young leaves, and root meristem is subsequently produced at their opposite poles. Therefore, *D. catenatum* roots do not originate from radicles, but from *de novo* root organogenesis, which may reflect the characteristics of the ancestors. The protocorm can not only protect the shoot body, store the nutrient starch, but also provide a habitat for the symbiotic microbe, establish a long-term and solid symbiotic relationship, and promote nutrient supply and stress tolerance.

*D. catenatum* root structure includes velamen, exodermis, cortex, endodermis, and stele. The exodermis and endodermis serve as two lines of defense to control the entry of substances, and the exodermis is more developed, which may be related to the requirement for high-efficiently filtering a large amount of water that are instantaneously captured by the velamen and preventing moisture escaping from the cortex. Thus, the cooperative evolution between velamen and exodermis confers aerial root of epiphytic orchid high tolerance to drought stress. In the cortex, water and minerals bind with storage substances to reside within the cell, bind with pectin polysaccharides to reside in the apoplast, and then further permeate through endodermis and enter the vascular system to transport.

The root development in *D. catenatum* is not only regulated by intrinsic genetic cues, such as germplasm, seedling age, and sampling segment, but also displays corresponding structural adaptability under different cultivation modes, especially for the velamen. When the root was grown in the matrix, the cells of each velamen layer were uniform in size; when exposed to the air directly, the outermost layer of velamen cells widened significantly; the cells closely attached to the bark still maintain viability, which are small and densely arranged. Whether root hairs are produced on the surface of the velamen depends on the availability and efficiency of nutrient acquisition. The roots exposed directly to the air or far from the pine bark substrate do not need to produce root hairs, whereas roots attached to the pine bark substrate or grown in agar medium can expand the absorption area by producing root hairs. Therefore, epiphytic orchid roots have evolved to make extensive elastic adaptations to environmental changes with economical and efficient principles. Under artificial aseptic culture conditions, the seed germination and seedling growth in most of plants can be directly cultured on hormone-free medium, but the seed germination and subsequent growth of *D. catenatum* require additional auxin application (1–2 mg⋅L^–1^ NAA); otherwise, the seedling becomes weak, indicating that the auxin synthesized by itself cannot meet its own needs. However, in the case of facility cultivation using pine bark substrates, *D. catenatum* grows normally, which may be related to the extra auxin supplied by its symbionts. A wide variety of orchid endophytic bacteria and fungi have been identified to promote plant growth *via* auxin secretion during seedling acclimation and root colonization (Faria et al., 2013; Pavlova et al., 2017; Shah et al., 2018, 2021, 2022; Chand et al., 2020). Therefore, preparing suitable concentration of auxin solution or isolating the auxin-producing symbiotic microorganisms can be directly applied to further improve the production and breeding of medicinal orchids.

It is known that root is usually very sensitive to auxin, and the lowest concentrations of indole-3-acetic acid (IAA) to inhibit the growth of root, shoot, and stem are around 0.01, 1 (=0.175 mg L^–1^), and 10 mm, respectively (Thimann, 1938). In Arabidopsis, 0.1 mg L^–1^ NAA has severely inhibited root growth, and 1 mg L^–1^ has completely abolished root growth (Supplementary Figure 2). However, the root of *D. catenatum* has high tolerance to auxin and grows best at the concentration of 5 mg L^–1^ NAA in this study. We speculated that the high tolerance of *D. catenatum* root to auxin is related to both the barrier function of the developed exodermis and osmotic regulation of storage matter in the cortex. Phylogenetic analysis suggests that IAA biosynthesis evolved independently in bacteria, fungi, and plants; IAA might serve as a widespread signaling molecule that mediates interspecies communication during evolution (Fu et al., 2015). Therefore, the biological significance of the high auxin tolerance of *D. catenatum* may be reflected in its interaction with external microorganisms, which helps to create a microhabitat preferring probiotics and inhibiting pathogens. For instance, high level of auxin treatments (10 and 100 mg L^–1^ IAA) considerably promotes the growth of several IAA-producing bacteria associated with *Dendrobium moschatum* (Tsavkelova et al., 2007). Exogenous application of 200 μm IAA inhibits the growth of some plant-associated pathogens, such as *Agrobacterium*, *Erwinia*, *Pseudomonas*, and *Xanthomonas* genera (Liu and Nester, 2006).

Exogenous auxin treatment on *D. catenatum* seedlings exhibited two effects: (i) Relatively low level of auxin promoted rooting and root elongation. Transcriptome analysis showed that the expression of root initiation-related genes, such as WOX family members, was not significantly changed, which may be related to the absence of lateral root in *D. catenatum*. However, cell wall synthesis-related genes (CESs and CSLs) were significantly upregulated, indicating that they play a role in root elongation. (ii) High level of auxin promoted root thickening and overproliferation. Anatomical observation showed that the differentiation of velamen and exodermis was inhibited, and the cortical parenchyma cells proliferated drastically, indicating that high concentrations of auxin can inhibit cell differentiation and promote cell division, which is consistent with the application of high level of auxin to induce explant dedifferentiation and callus formation in tissue culture. Correspondingly, the genes related to stem cell control and regeneration pathways were significantly upregulated, especially WOX11-WOX5 root initiation programs, in line with the established rooting pathway during callus induction (Sugimoto et al., 2010; Hu and Xu, 2016); cell division regulatory genes (*CYCA1;4*, *B1;1*, *D2;1*, *D3;2*) were also significantly induced, consistent with the rapid proliferation of cells.

In Arabidopsis, WOX11/12 are involved in accelerating the formation of AR or/and callus from explants in the presence of auxin (Liu et al., 2014). In rice, OsWOX11 participates in shoot and root development. Both os*wox11* mutant and *OsWOX11* overexpression rice display reduced shoot growth. Moreover, blocking of *OsWOX11* also inhibits crown root development, and overexpression of *OsWOX11* accelerates crown root cell division and dramatically shoot-borne crown root production (Zhao et al., 2009, 2015; Cheng et al., 2016, 2018). Here, overexpression of *DcWOX12* in Arabidopsis inhibits chloroplast and leaf differentiation and reduces root length and number of lateral roots, which is different from Arabidopsis and rice *WOX11/12* overexpression phenotype. Intriguingly, *DcWOX12* overexpression can directly form highly proliferative callus in the absence of auxin, displaying stronger cell dedifferentiation and regeneration ability than *WOX11*/*12* homologs in Arabidopsis and rice, and somewhat reminiscent of overexpression of SAM master regulator *WUS* (Zuo et al., 2002; Gallois et al., 2004), which provide an exciting new gene resource in breakthrough of high-efficient monocot genetic transformation method in a broad range of genotypes and species (Hofmann, 2016; Lowe et al., 2016). Transcriptome sequencing showed that stem cell control, regeneration pathway, and embryo-specific gene expression were significantly increased in *DcWOX12* overexpression plant. Thus, *WOX11/12* homologous genes in different species not only retained a conservative role, but also underwent functional divergence during evolution. In addition, overexpression of *OsWOX11* in rice improves drought resistance by modulating rice root system development (Cheng et al., 2016). Specific expression of *OsWOX11* in rice roots greatly improves root growth and activity and results in increased K uptake and grain yield in low K soil (Chen et al., 2015). Overexpression of *PagWOX11/12a* enhances salt and drought tolerance in poplar (Wang et al., 2020, 2021). Therefore, different WOX11/12 versions from different species could be exploited in biotechnology to improve plant resistance to abiotic stresses *via* manipulating plant root development and to improve the efficiency of monocot plant transformation *via* promoting cell dedifferentiation and cell fate transition.

## Conclusion

This study suggests unique developmental characteristics of *D. catenatum* aerial root closely associated with its high environmental adaptability to special epiphytic life mode. Furthermore, *D. catenatum* root displays high tolerance and dual responses to auxin, possibly also related to its adaptability. Heterozygous overexpression system indicated that *D. catenatum* WOX12 confers cell high-efficient pluripotency acquisition properties. Therefore, this study provided not only an insight into ideal root structure breeding of simulated natural cultivation in *D. catenatum*, but also a novel target gene for improving the efficiency of monocot plant transformation, especially in orchid plants.

## Data Availability Statement

The original contributions presented in this study are publicly available. This data can be found here: NCBI, PRJNA815882.

## Author Contributions

DC and CL planned and designed the research. JT and WJ performed the experiments. DC, JT, CL, ZH, and WJ analyzed the data. DC and JS wrote the manuscript. All authors approved the manuscript.

## Conflict of Interest

The authors declare that the research was conducted in the absence of any commercial or financial relationships that could be construed as a potential conflict of interest.

## Publisher’s Note

All claims expressed in this article are solely those of the authors and do not necessarily represent those of their affiliated organizations, or those of the publisher, the editors and the reviewers. Any product that may be evaluated in this article, or claim that may be made by its manufacturer, is not guaranteed or endorsed by the publisher.

## Footnotes

1
http://www.plantprobes.net/

2
http://www.omicshare.com/tools

## Supplementary Material

Supplementary Figure 1 Phylogenetic analysis of WOX proteins from *D. catenatum* and Arabidopsis. The phylogenetic tree was constructed according to the NJ method by MEGA 7.0 with 1000 bootstrap replicates.

Supplementary Figure 2 Effect of auxin on Arabidopsis root development. Arabidopsis was grown in MS medium containing 0, 0.1, 0.5, and 1 mg L^–1^ NAA, respectively. Bars = 1 cm.
