# Supplementary figures and images for: Developmental Characteristics and Auxin Response of Epiphytic Root in Dendrobium catenatum
Source: Front Plant Sci. 2022 Jun 23;13:935540. doi: 10.3389/fpls.2022.935540 (PMC9260429; doi:10.3389/fpls.2022.935540)

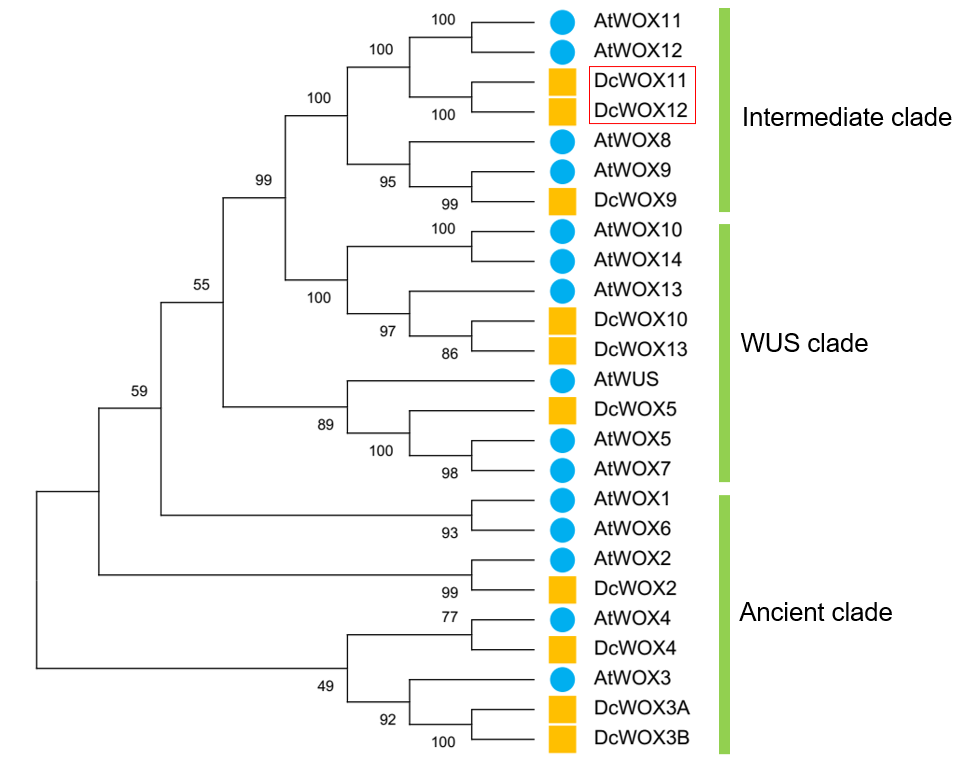

Supplement: Supplementary Figure 1 — Phylogenetic analysis of WOX proteins from D. catenatum and Arabidopsis. The phylogenetic tree was constructed according to the NJ method by MEGA 7.0 with 1000 bootstrap replicates. [file Image_1.PNG]

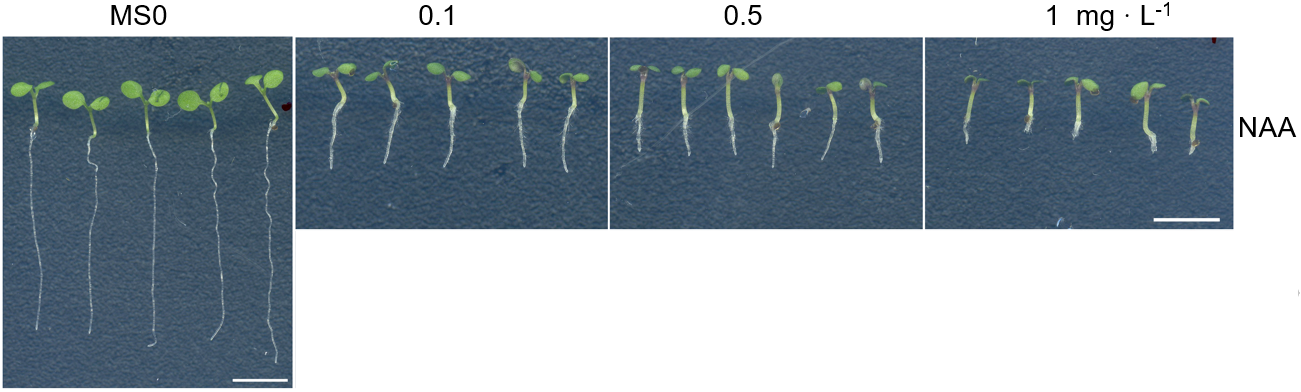

Supplement: Supplementary Figure 2 — Effect of auxin on Arabidopsis root development. Arabidopsis was grown in MS medium containing 0, 0.1, 0.5, and 1 mg L–1 NAA, respectively. Bars = 1 cm. [file Image_2.TIF]
